# Prisons, Older People, and Age-Friendly Cities and Communities: Towards an Inclusive Approach

**DOI:** 10.3390/ijerph17249200

**Published:** 2020-12-09

**Authors:** Helen Codd

**Affiliations:** School of Justice, University of Central Lancashire, Preston PR1 2HE, UK; hlcodd@uclan.ac.uk

**Keywords:** prisons, prisoners, older offenders, ex-prisoners, age-friendly cities and communities

## Abstract

This original and ground-breaking interdisciplinary article brings together perspectives from gerontology, criminology, penology, and social policy to explore critically the nature and consequences of the lack of visibility of prisons, prisoners, and ex-prisoners within global research, policy and practice on age-friendly cities and communities (AFCC), at a time when increasing numbers of people are ageing in prison settings in many countries. In addition, the COVID-19 pandemic continues to pose challenges in the contexts both of older peoples’ lives, wellbeing, and health, and also within prison settings, and thus it is timely to reflect on the links between older people, prisons, and cities, at a time of ongoing change. Just as there is an extensive body of ongoing research exploring age-friendly cities and communities, there is extensive published research on older people’s experiences of imprisonment, and a growing body of research on ageing in the prison setting. However, these two research and policy fields have evolved largely independently and separately, leading to a lack of visibility of prisons and prisoners within AFCC research and policy and, similarly, the omission of consideration of the relevance of AFCC research and policy to older prisoners and ex-prisoners. Existing checklists and tools for assessing and measuring the age-friendliness of cities and communities may be of limited relevance in the context of prisons and prisoners. This article identifies the potential for integration and for cross-disciplinary research in this context, concluding with recommendations for developing inclusive research, policies, and evaluation frameworks which recognise and include prisons and older prisoners, both during and after incarceration.

## 1. Introduction

### 1.1. Age-Friendly Cities, Crime, and Criminology

The development of “age-friendly cities and communities” (AFCC) has become a highly significant theme in relation to public policy and ageing that has resulted in a Global Network for Age-Friendly Cities and Communities, launched in 2010, and a panoply of locally-developed age-friendly policies [1]. When engaging with the research literature for the first time, however, an academic working on crime and justice issues is likely to be struck by a vague sense of cognitive dissonance. Age-friendly cities are promoted as spaces, places, and communities where people of all ages are valued, engaged, and facilitated to live active lives. This overarching vision of the age-friendly city portrays a positive image of a harmonious, inter- and multi-generational space, place, and community where all are welcomed and included. However, for criminologists, cities have always been linked to crime, urban deprivation, and a range of social issues including drug use, organised crime, homelessness, “gangs” and high levels of both criminal residence and criminal victimisation.

That is not to say, of course, that criminological research has not identified positive aspects of cities—there is a substantial body of research on community cohesion in cities, and on designing cities to encourage crime prevention—but from the early days of the pioneer researchers of the Chicago School, who used the city of Chicago as their laboratory for empirical social research, there has been no shortage of research exploring crime, policing and victimisation within urban environments, although a detailed exposition of this vast body of research is beyond the scope of this article [2,3].

Thus, on encountering research and policy documents on age-friendly cities and communities for the first time, a criminologist is very likely to wonder where the crime has gone, and indeed, where all the criminals have gone. Indeed, from a more penological perspective, one could also wonder where the prisons and ex-prisoners have gone, especially when, in the UK and many other countries, prisons situated in urban environments are still common. That is not to say there is no mention of crime at all, as in some of the research there is some mention of policing and older people as victims, for example, but the relative invisibility of prisons, older offenders, older prisoners, and former prisoners is striking. This reflects the relative neglect of older peoples’ experiences as offenders, prisoners, and ex-prisoners within gerontology as a whole, although a recent edited collection exploring diversity and difference in experiences of ageing included a chapter on ageing in prison, and this inclusion is to be welcomed [4].

To some extent this is surprising, as the evolution and development of the “age-friendly cities and communities” (AFCC) movement from the early 1980s onwards has been mirrored by a growing recognition of the involvement of older people in the criminal justice process, as victims but also as offenders. This growth in awareness and expansion in the published research literature reflects both demographic shifts, with older people living longer, and also changes in penal policies and practices which have led to high levels of imprisonment in some jurisdictions. This “mass imprisonment epidemic”, perhaps most visible in the US but replicated to some extent in other countries including England and Wales, has been characterised by mandatory minimum sentencing, whole life tariffs, and life sentences without the possibility of parole. The abolition of capital punishment in many countries has also meant that individuals are growing old in prison when prior to abolition they would have been executed. In addition, as seen in recent cases in the UK and the US, prosecutions of historical state, war, and sexual crimes which took place in the past are leading to people who are now in older age being tried and sentenced, sometimes with no previous experience of involvement in the criminal justice system. However, these two fields of research have emerged and evolved largely independently of each other and whilst there is a great deal of potential for exchange of good practice at present these two disciplinary approaches seem to constitute primarily separate academic and policy-making spaces.

### 1.2. The Aims and Structure of This Paper

The aims, objectives, and purposes of this review are to explore the extent to which the needs and experiences of older prisoners and former prisoners are recognised within the extensive and growing literature on age-friendly cities; to assess the relevance of existing measures of age-friendliness in relation to older prisoners and former prisoners, and to identify gaps in the literature and outline directions for future research. This paper is based on a thorough and comprehensive literature review including not only monographs and peer-reviewed articles but also relevant reports by non-governmental organisations (NGOs) and governmental policy guidance documents.

The structure of this paper reflects the potential readership which includes academics and policy-makers with expertise in criminology and also those with expertise in relation to age-friendly cities. Whilst some readers will approach this paper with some knowledge of older prisoners, they may not be familiar with the literature on age-friendly cities, and the converse may also be the case. Recognising this diverse potential readership, Section 2 of the paper outlines key issues identified in the published research into older people and prisons. Section 3 provides a summary of the development of the concept of age-friendly cities and communities (AFCC), including an introduction to the eight WHO themes. Section 4 brings these two research areas together to explore the age-friendliness of cities and communities for older prisoners and ex-prisoners, using the eight WHO themes as a starting point. Section 5 sets out conclusions, including recommendations for future research.

## 2. Older People and Prisons

### 2.1. The Rising Number of Older Prisoners

In many countries including the UK, Ireland, the US, Canada, and Australia, older prisoners, make up a significant and growing minority within a penal estate populated primarily by young men [5,6,7,8]. Rising numbers of older prisoners have become a matter of concern for policy-makers, practitioners, and researchers in many jurisdictions including the US, the UK, and Japan. Although these numbers are increasing, older prisoners form a minority of the prison population, and within this population older women constitute a minority within a minority [8].

### 2.2. The Experiences of Older Prisoners

Alongside the “greying” of the prison population, research has flourished and there is now a substantial body of relevant literature, utlilisinga range of quantitative and qualitative methodological approaches, which have served to render older prisoners and their experiences and needs much more visible in criminological and penological contexts. This research includes perspectives from law, psychiatry, psychology, medicine, health and gerontology, sociology, social work, social and penal policy, criminology, corrections, and prison management [6,7,9,10,11,12,13,14,15,16,17,18,19,20,21,22,23,24]. There is also an extensive body of research on criminal behaviour by, and against, older people, discussion of which is beyond the scope of this article [10,25].

The term “prison” is used in this article in the usual UK sense, meaning an establishment tasked with the custodial care of those accused of crimes awaiting trial or sentence, and those who have been sentenced to terms of imprisonment (or youth custody for young offenders). This includes a wide range of institutions, including the equivalents of jails and also the equivalents of federal penitentiaries, as exist in the US. Prisons are not homogenous environments, but they all involve compulsory detention and associated restrictions on liberty. They vary not only in terms of their physical environments and facilities, but also their populations: for example, a city-centre Victorian prison such as HMP Preston, is populated mainly by remand prisoners and experiences a high level of prisoner turnover (“churn”) and an unstable and ever-changing population, with security appropriate to holding Category B prisoners. This can be contrasted with long-term training prisons and contrasted again with the highest security “dispersal” prisons (such as HMP Long Lartin) and prisons operating under open conditions (such as HMP Kirkham). Some prisons have higher proportionate numbers of older offenders as a reflection of their specialist status as exemplified by HMP Wymott and its high population of sex offenders. All prisons in England and Wales are subject to the same inspection regime but these same inspection reports illuminate differences between establishments.

Prisons fulfil multiple functions for those who live in them due to their sentence. They provide bed, board, and a place to live. They are the source of medical care, of education and training and for some prisoners very low-wage employment. They can provide psychological care, counselling, and spiritual support and opportunities to practise one’s faith (or not). The prison can create new friendships but also expose older people to aggression and bullying. Prison can entrench people in offending lifestyles, as reflected in reoffending rates. From the point of view of an older prisoner, the prison fulfils multiples roles, often at the same time. Some of these mirror outside activities and provision. Others make “ordinary” life challenging, such as restrictions on family visits and contact. Access to telephones and the internet may make older people’s lives easier in the community, but their use in prison is highly proscribed. The COVID19 pandemic has created a necessity for new forms of communication between prisoners and their friends and family, including approved prison-issued mobile phones and online-based “Purple Visits”, but these are tightly regulated and, at the time of writing, subject to technological and infrastructural uncertainties and the challenges of digital poverty.

### 2.3. The Age-Friendly Prison?

There are ongoing debates about if and how prisons can be age-friendly institutions, especially as a core element of age-friendliness involves maintaining autonomy and choice and it is inherent in the nature of the prison itself that such autonomy and choice is restricted. The most straightforward approach to age-friendliness in prisons is to mirror the “-friendly” suffix from other aspects of prison provision, such as prisons being “family- friendly” or “child-friendly”, then applying these ideas to exploring whether prisons respond adequately to the needs of prisoners from diverse age groups, primarily in the context of ageing (and elderly) prisoners. Applying this approach, there is an array of published research which highlights failures to be age-friendly, especially in relation to physical factors such as prison design, medical, and healthcare facilities, and which documents the challenges experienced by older prisoners [6,20,21,26]. To some extent the literature on older prisoners has, until relatively recently, tended to view older people in prison through a medicalised lens. More broadly, older prisoners are also included in research on adult social care in prison settings [26,27].

### 2.4. Good Practice in Working with Older Prisoners

RECOOP, an organisation which supports older prisoners, has published guides to good practice in working with older prisoners, and also for approved premises [28]. There is no shortage of research literature criticising prison environments for the barriers to participation they create for older offenders, especially those with mobility difficulties, and attention has been drawn to access to educational and dining areas; problems of allocating older people the top bunk of bunk beds; sports and recreation facilities, and the limitations to access and movement inherent in old prison buildings, many of them, such as HMP Dartmoor, built initially in the nineteenth century. Some attention has been drawn to the social needs of older prisoners but much of the research focuses on physical and environmental factors, and issues of provision to respond to the medical, health and welfare needs of older people in prison. This approach focuses on a medicalised, pathological model of ageing and to some extent focuses on micro-environmental factors (grab rails, ramps, and so on) which are relatively low-cost to amend and which do not require major changes to prison regimes, provision of activities, and a whole scale rethinking of prison for older people [6,12,18,21].

There are also several policy reports and guidance documents aimed specifically at prison governors and service commissioners to help them to respond to the needs of the older prisoner population within prisons, as exemplified in HMPPS Model for Operational Delivery: Older Prisoners [29] and the work of the Prisons and Probations Ombudsmen [30]. Similarly, research reports and policy documents from government agencies and NGOs provide information and guidance on specific aspects of the needs and experiences of older prisoners, including health and social care needs [27,31,32] and issues arising from the needs of older people on release [33]. The ageing of the prison population has also prompted discussions around life-limiting illness and end-of-life care, including palliative care, the ethical challenges and issues around assisted suicide and assisted dying, and the impacts of the deaths of loved ones during the period of custody [34,35,36,37].

### 2.5. The Challenges of Defining “Older” in the Prison Setting

Older prisoners are no longer “invisible” in penological research and policy development, nor conversely are older people in prison completely invisible in research on ageing, but in spite of the expansion of academic and practitioner interest in older offenders a number of core questions still vex researchers. From the outset, the definitions of “older people” and “older prisoners” have been contested, and there is no agreed national or international definition, each researcher or policymaker adopting their own definition which sets the threshold somewhere between 45 and over 70 [38]. Although the UN has recognised older prisoners as “special needs prisoners” [39] there is no shared international definition, which makes comparisons difficult, and research tends to use the terms “older” and “elderly” interchangeably [13]. Defining people as older at 45 or 50 may seem very low, but it has been argued that accelerated aging can occur for some prisoners, who are argued to be functionally older than their chronological age [19] as a consequence of previous lifestyle, lack of medical care prior to imprisonment, and the experience of incarceration itself [13,14,15]. Thus, a prisoner in their fifties may have the health problems and physical appearance of someone living in the community who is at least ten years older [9,10,37]. This view is controversial, however, some commentators arguing that prison healthcare can mitigate the accelerated aging process and that individuals experience aging differently [38]. 

## 3. Age-friendly Cities and Communities

### 3.1. The Development of Age-Friendly Cities (AFCC)

The AFCC movement traces its origins to the United Nations First World Assembly on Ageing, which was convened by the General Assembly of the UN and held in Vienna in 1982 [40,41,42]. Subsequent to this conference, which led to the first ever international instrument on ageing (the Vienna International Plan of Action on Ageing), the 1986 WHO Ottawa Charter for Health Promotion led to the launch of the Healthy Cities movement. In 2002, twenty years after the First World Assembly, the UN met to review the outcomes of the Vienna International Plan on Ageing. This 2002 event led to the adoption of two major policies which provided the foundation for the AFCC movement (i.e., The Madrid International Plan of Action on Ageing and the WHO Active Ageing Policy Framework. The WHO defined an age-friendly city as one which could promote active ageing, defined as, “the process of optimizing opportunities for health, participation and security in order to enhance quality of life as people age” [42].

The age-friendly city programme, having first been introduced at the World Congress of Gerontology and Geriatrics, was launched in 2006, as the WHO Global Age-Friendly Cities project, which brought together 33 cities around the world in order to identify the core features of an age-friendly city. This research, which focused on the viewpoints of older people, caregivers, and local service providers, identified eight themes in order to increase and maximise the age-friendliness of cities, each including a checklist of key features. This guide and checklist aimed to provide cities with a tool to identify strengths and areas for improvement, to plan change and to monitor progress [43,44].

The WHO is not the first, nor indeed the only, organisation to focus on age-friendly developments, but the WHO has become a highly significant and influential resource for defining AFCCs [45]. Other initiatives have been developed, including the creation of an Action Group by the European Commission, which more recently has created a joint project (Age-Friendly Environments in Europe (AFEE)) with the WHO Regional Office for Europe; the AARP livable communities approach; the AdvantAge Initiative created by the Visiting Nurse Service of New York, the US Environmental Protection Agency’s Building Healthy Communities for Active Aging (BHCAA) Award Program, and the “Village Movement”, among others. The WHO has facilitated links, support and dialogue between different cities, communities, and regions, via a global network, and the identification of the eight domains has encouraged and enabled an integrated approach to ageing and urbanisation which goes beyond health and social care programmes alone [42]. Beyond urban environments, age-friendliness has been applied to exploring the lived experiences and social exclusion of older people in rural environments [44].

The concept of the “livability” of cities predates research and policies exploring AFCCs [45]. Terms such as livability and “age-friendliness” are used to describe how cities and communities are recognising and responding to the needs of an ageing population, often being used interchangeably. However, these concepts emerged at different times. Livability emerged during the 1980s in relation to cities and can include but is not limited to ageing people within cities. In a significant and useful scoping review, Chonody and Teatra [45] utilise a five-step process to explore the similarities and differences between these approaches, exploring whether they are underpinned by a similar perspective. Their review explores how livable and age-friendly communities are conceptualised in the existing literature, the specific elements that are identified as formulating the concepts, such as frameworks or indices, and the extent to which these frameworks and indices are interrelated and/or independent. Their thorough methodological approach identified 21 studies which met their criteria for inclusion, which were analysed in order to identify the elements of livable and age -friendly communities in each, that were generated through along with the frequency of their occurrence over all the studies included.

Chonody and Teatra’s article provides several tables identifying the core terms and themes used within each study to identify livability and age-friendliness. The amount of variation as to how these themes are defined and framed is striking. Overall, however, they find several overlapping and common thematic frameworks, such as health, social engagement/connectivity, opportunities for recreation, and employment or volunteering opportunities. The most frequently included community elements were health, housing, safety and security, social participation, transportation, civic participation, the built environment, recreation and cultural activities, the natural environment, income, and respect or social inclusion. Some of the definitions of livability, those which considered citizens’ inclusion, included age-friendliness. Overall, the age-friendly frameworks were more focused on ageing and ageing populations in comparison with the livability frameworks which seemed in some settings to be more directed towards young professionals [46]. As Kashef [47] pointed out, “The cities that tend to rise to the top of the livability rankings are those with relatively small percentages of people living below the poverty line, low birth rates, low percentage of children, and are more hospitable to tourists/business travelers than immigrants.” If, however, livability is assessed from the points of view, for example, of those people who are from a lower socioeconomic status, then this highlights gaps in services and structures.

### 3.2. The Eight WHO Themes of Age-Friendly Cities

The eight WHO themes have become one of the most commonly used tools for evaluating age-friendliness in varied environments around the world [48] following the publication of the WHO guide, “Global age-friendly cities” in 2007 [42]. The eight themes explored in the guide are:

1. Outdoor spaces & buildings
2. Transportation
3. Housing
4. Social Participation
5. Respect and social inclusion
6. Civic participation & employment
7. Communication and Information
8. Community support and health services

Within research and policy documents on age-friendly cities and communities, it is a foundational principle that every community and city will have its own unique challenges and opportunities to address, the WHO encouraging each community and city to implement evidence-based planning and to develop their own mechanisms in order to increase their age-friendliness A number of toolkits, resource packs and guides have been developed so as to provide a basic understanding of age-friendly cities, some providing templates to help cities assess and evaluate the effectiveness of their programmes across each of their domains [48,49]. The challenges of measuring age-friendliness have been recognised by the WHO itself—”age-friendliness is a moving target; thus, it does not easily lend itself to standardization of measurement” [50]. From criticisms that initial frameworks for assessing and evaluating age-friendliness, the WHO has published further core and supplementary AFC indicators, some of which are very specific [49].

The first step as encouraged by the WHO is to conduct a baseline assessment of age-friendliness, such as by consulting with older people, organisations, and other stakeholders via focus groups or more innovative participatory methods such as walking interviews or co-produced research involving older people [48] Once these priorities and goals have been identified then cities are encouraged to devise their own action plans and monitor the subsequent implementation and progress of activities and initiatives [42]. Throughout these plans there is a focus on developing partnerships and collaborative working; involving older people in the decision-making process; and monitoring progress and evaluating the results. 

## 4. Prisons and Age-Friendly Cities and Communities: Research, Policy, and Practice

### 4.1. Prisons as Urban Institutions

The published literature on age-friendly cities does not usually recognise prisons as significant urban institutions comparable, for example, with hospitals, shops, and leisure facilities, even though they may be large, visible buildings employing a significant local workforce. When prisons are physically and geographically located away from city conurbations, they are linked to cities and city communities via the backgrounds and relationships of prisoners, along with other individuals and organisations which engage with the prison and its residents. The city may be the home residence area of prisoners, or the locale in which their offending has taken place, or the area to which they are likely to be released. For some former prisoners, the city becomes their residence after release simply because they are unable to return to their previous home addresses, towns, or regions, sometimes because of the impact of their offending, associated stigma, or for the protection of the public, their victims or themselves.

The interactions between prisons, cities, and communities manifest themselves in a variety of ways. Prisoners themselves may be located in one geographical location (the prison); originate in a different location (e.g., their “home town” or city) and then engage with and be visited by people living in other places entirely. The nature of the isolation of prisoners from local communities, unless they are in open or semi-open conditions where, for example, they are allowed out to work or on other forms of release on temporary license (ROTL), means that they “belong” more to their home locale than where they may live, sometimes for many years.

### 4.2. Ageing, Prisons, Families, and Friends 

When we discuss prisons, prisoners, and age-friendly cities and communities it is important to include non-imprisoned older people who, although they have not themselves been convicted, live within the “web of imprisonment” through their relationships with prisoners [51]. This can include most obviously the partners and spouses of prisoners, but can also include parents, children, other relatives and kin, and friends. As older offenders are themselves experiencing ageing, and demographic changes mean they are living longer, so may be their parents, meaning that older prisoners may themselves be visited by people who are even older. This is particularly significant in the light of the research into people who commit serious offences which attract high levels of media attention, such as some homicides and sexual offences, when after intimate and romantic relationships have ended, parents, especially mothers, continue to support their incarcerated adult (and elderly) child [51]. Thus, in the hypothetical situation of a man being convicted of multiple rapes and murders at the age of thirty, and being told at sentencing he will be subject to a whole-life tariff and die in prison, if his mother was 55 when he was thirty then by the time he is 60 his mother would be 85, and if he lives until he is 75 then his mother would be 100. Mothers are more likely than other friends or family members to continue to support their imprisoned adult children, and in most countries, women live longer than men, and thus this situation is not uncommon. If the prison is not conceptualised as part of the age-friendly city or community then these challenges experienced by non-imprisoned older visitors may go unnoticed.

### 4.3. The WHO Domains and Older Prisoners

The eight domains of age-friendliness set out by the WHO provide a useful starting point for exploring the age-friendliness of cities and communities for older prisoners and ex-prisoners. 

#### 4.3.1. Outdoor Spaces and Buildings

The first theme, that of age-friendliness of outdoor spaces and buildings, may not pose additional challenges beyond these experienced by non-imprisoned older people. However, criminal justice system agencies, including the police, probation, and other services for offenders, need to be as accessible to older people as younger people, even though the age profile of their client group may be younger.

#### 4.3.2. Transportation

If the prison is located in a central urban setting then, as long as public transport is accessible for older people, or there are specific community transport resources which can be used, then visiting the prison may not pose a great transport challenge. However, if there is little availability of relevant transport, and the prison is, like many prisons, built on the edge of a city or, as in the US and some parts of the UK, in a rural area, then the problems of transportation to prisons for family members, as already identified in the literature on prisoners’ families, may be magnified where older people are experiencing mobility difficulties, chronic illnesses and mental health issues, including dementia. Health challenges may make long journeys impractical or painful. The transport problems of visiting prisons in the UK are well-documented, and then if we add in the challenges of ageing then the problems may be magnified, and also have a disproportionate negative impact on members of the poorest communities, who may not have access to cars or be able to drive. We already know that in the UK female prisoners are held further away from their homes and families than male prisoners, and thus these transportation difficulties can be compounded.

#### 4.3.3. Housing 

For older people leaving prison, age-friendly appropriate housing may be difficult or impossible to access. Where individuals have aged in prison, their partners/spouses may have died or relationships broken down; children may have grown up, and the “family home” if it ever existed at all, may not be there as a place to which the released prisoner can return. There is also the core problem of the concept of “ageing in place” and what happens to people who have either never had a “place” in which to age, if they were previously homeless, for example, or where their place to age was the prison. As a carceral setting the prison is not intended to offer a long-term residential placement where prisoners’ sentences have ended, or where their risk assessment indicates that parole/release on license is appropriate, but the question then is where they should go.

Residential care homes may not be willing or able to house ex-prisoners, and designated residential settings for ex-prisoners (such as approved premises in the UK) may be unsuitable as they are populated mainly by younger men and may not have appropriate care/nursing facilities or create opportunities for bullying and harassment. Older ex-prisoners may thus not be able to be housed within adult social care settings for older people, nor within criminal justice/probation settings. That said, in Canada, Haley House in Peterborough ON is a pioneering unique halfway house for formerly imprisoned older men run by a non-profit organisation and funded mainly by the Correctional Service Canada (CIC) [50]. Older people leaving prison who do not have specific care needs face difficulties accessing public and private housing due to background checks and the need for references, and those convicted of sexual offences face additional barriers to accessing housing. These barriers create a risk of homelessness, and on becoming homeless, may not be able to access homelessness services because of their criminal convictions and/or risk profile. For example, older people who are homeless after prison may find that even if they previously accessed homelessness services once they have become older these services may not be suitable for them and specific provision for older people who are homeless is very rare [52]. Where older age intersects not only with a prior record of crime and incarceration but also mental health issues and substance abuse, street homelessness may be experienced as inevitable due to a lack of appropriate accommodation and services, even though lack of a settled address creates additional challenges of offender and risk management [53]. Homelessness itself is a traumatic event and creates additional risks of victimisation, including violence [54]. Although nearly all of the published research focuses on formerly imprisoned men, older female former prisoners constitute a subgroup of a subgroup, or a minority within a minority, which is even more vulnerable [53].

#### 4.3.4. Social Participation

Social participation is interlinked with social integration, which has been recognised as a very significant element of the process of release and successful community resettlement and reentry. Older people may well be at risk of social isolation and loneliness after release, not only because of stigma and hostility, but also because if they have aged in prison then they may no longer have any supportive family or friendship networks which can be beneficial in relation to the practical and emotional impacts of release and resettlement. Fear of hostility, and fear of stigmatisation, can be powerful behavioural influencers in themselves, even if there are no actual hostile incidents or interactions. Family ties may never have existed, or could have been lost as a consequence of the offending or the sentence, with older offenders sometimes severing ties themselves. Strong family ties have been linked to increased levels of desistance (for all age groups) and lower levels of reoffending: older offenders may not be able to benefit practically, mentally, or emotionally on release, and thus may experience high levels of perceived disconnectedness [55]. Loneliness is a common experience for many older people, and this is exacerbated by the stigma of a criminal conviction and prison sentence, along with offender management controls over managing risk by defining, for example, where people can go and when, and with whom they can (or cannot) associate.

The same challenges, marginalities and invisibilities can apply too in relation to non-residential settings and community programmes. There are related issues around long-term prisoners and institutionalisation: long periods in prison create challenges to community re-entry, and these are magnified for older people. 

#### 4.3.5. Respect and Social Inclusion

Respect and social inclusion are linked to social participation. To sentence someone to prison is inherently an act of social exclusion, a symbolic denunciation of someone’s conduct and a statement that a person is not welcome within the community. The status of offender, especially that of a prisoner or ex-prisoner, is usually a stigmatised identity and means that prison itself denotes someone as undeserving of respect, beyond the basic requirements of respect for human rights. Whilst older offenders are not all sex offenders, a substantial proportion are, and risk management and safeguarding may make some forms of social participation impossible due to risk management considerations. For example, if a community wants to create opportunities for inter- and multi-generational engagement, bringing children into contact with an adult convicted of sexual offences against children can be very dangerous. 

#### 4.3.6. Civic Participation and Employment

In the UK, despite several challenges via the European Court of Human Rights, prisoners serving a custodial sentence after conviction are not eligible to vote in any elections, echoing archaic historical concepts of “civil death” as a consequence of conviction. Civic participation is particularly challenging in jurisdictions, including some US states, where some or all ex-prisoners are disenfranchised, sometimes for life, by reason of a felony conviction. These felony disenfranchisement laws mean that not only are some convicted offenders banned from voting and banned from seeking civic and political offices, but there is no political advantage to be gained by politicians and civic leaders if they engage with the views and needs of ex-offenders and ex-prisoners [56].

Ex-prisoners of all ages face barriers to employment as a consequence of conviction and sentence. This can be linked directly to the nature of the offending, which may mean that some forms of employment are no longer available due to safeguarding requirements and criminal records checks, and also to the reluctance of many employers to employ ex-prisoners. Age discrimination is also a concern [57]. For older people leaving prison, unless they have a previous job to which they can return, or have the capacity to set up their own business or become self-employed, finding employment is often impossible, especially in times of recession where there is a very high level of competition for any available jobs [58,59]. 

#### 4.3.7. Communication and Information

Accessing information can be challenging for some older people leaving prison, especially as many governmental agencies and advice organisations have moved to online resources and delivery. This is even more visible due to the impacts of the COVID-19 pandemic, which has accelerated anticipated shifts towards online provision of information and advice. Governmental agencies (such as the DWP in the UK) require all new claims to be made via their online portal. However, older people leaving prison may struggle to access these portals, due to digital poverty and lack of access to smartphones and the internet, and also especially if they have been long-term prisoners, due to a lack of knowledge, skills, and experience of using IT technologies which have developed apace during their sentence. Prisons exercise strict controls on access to mobile telephones and the internet, and for some offenders post-release supervision requirements, such as specific offending and harm prevention orders for sex offenders, mean that accessing the internet is banned entirely.

#### 4.3.8. Community Support & Health Services

Older people who leave prison may be marginalised within the provision of community support services because they constitute a minority within the client group of an organisation. For example, criminal justice agencies, including probation, manage a caseload which is predominantly young and male. For organisations supporting older people, the particular needs of older people after leaving prison can present unfamiliar challenges, especially if offending behaviour co-exists with mental health issues, substance misuse, and homelessness. Older ex-prisoners fall through the gaps in service provision by virtue of being a minority within every client group, especially at a time when health and social services budgets have been cut drastically. 

## 5. Conclusions: Towards Inclusion

The invisibility of prisons and former prisoners within age friendly cities reflects the stigmatised identity of the prison as an establishment and as an institution, and ongoing stigmatisation of prisoners and ex-prisoners. At best, this reflects what Crawley [60] and Cadet [61] refer to as “institutional thoughtlessness” and at worst this reflects discrimination against older people who offend or have a history of offending. Research, practice and policies on Age-Friendly Cities and Communities promulgates a vision of a positive, friendly, accessible locale and community, including active, engaged, and participatory older citizens, and older ex-prisoners, especially those who become homeless or continue to offend, do not fit easily into this almost-utopian vision. The situation becomes even more complex for older ex-prisoners who carry multiple marginalised identities linked to poverty, mental health, race and ethnicity, sexuality, illness and disability, and offence type. In developing further research, policy and practice it is important to recognise and unpack diversity within and between older people, and not to assume homogeneity on the basis of age. Ageing intersects with other factors including ethnicity, gender, sexuality, and socio-economic status, although the nuances of how these factors influence experiences of imprisonment and release from prison are under-researched. 

The WHO domains are of some relevance to older prisoners and ex-prisoners, but further research is needed so as to identify the specific aspects of age-friendliness which are most important to older prisoners and older ex-prisoners. This research could also explore whether elements of the work of Buckner et al. [62] would be of value too. Buckner et al. [62] aimed to identify an evidence-based approach cities could use that (i) can be applied in different contexts, (ii) reflects the complexity of the initiatives, (iii) draws on sound data to make assessments of potential or demonstrable effectiveness, and (iv) presents findings clearly to a mixed audience. Their work presents an evaluation tool which responds to these requirements, based on fieldwork in Liverpool, UK. In contrast with the WHO indicators, which are often very specific, this tool is designed to gather evidence on a wider scale. It can indeed accommodate the WHO indicators, but these can be subsumed within the broader input areas, especially those of “provision and involvement of older people”. Buckner et al. [62] argue that applying the WHO indicators together with the tool can draw attention to very specific issues which would be less visible when assessed by the tool alone. 

**Figure 1:**
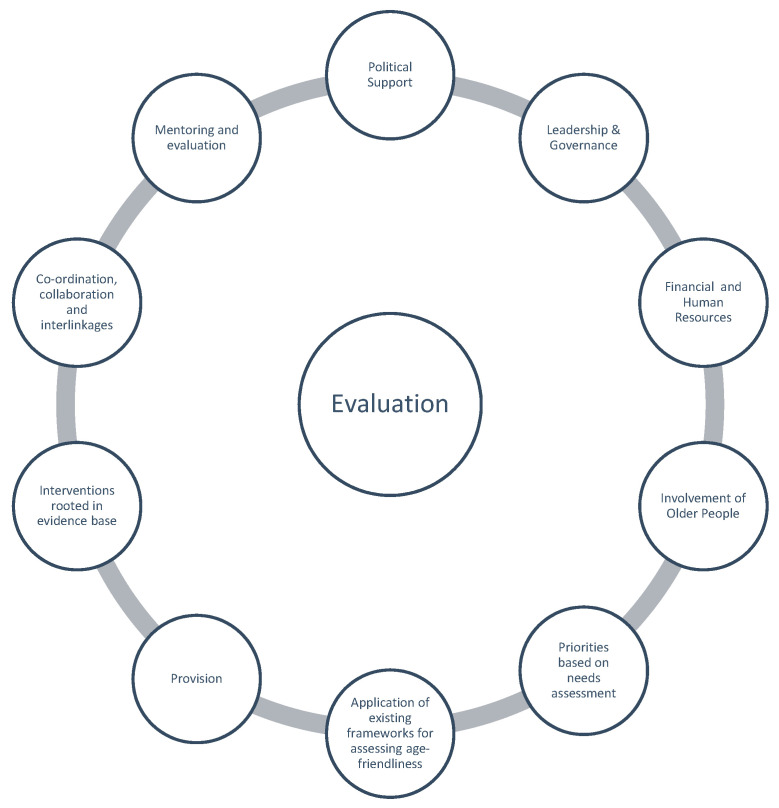
Buckner et al.’s input areas.

The evaluation tool developed by Buckner at.al. [62] evaluation tool includes ten “input areas” for which evidence is required to assess policy and practice initiatives which strive to be age-friendly. These input areas are shown in Figure 1.

In their conclusions they highlight the potential of the tool to act as, “an integrating framework for different city strategies that include an age-friendly agenda”. They recognise that this might include adapting the ten input areas so that they can act as a generic guide and assessment framework “for and across diverse strategies”, having discussed with stakeholders the potential relevance of the tool to other city-wide strategies including families, health promotion and housing.

With this in mind, future research which is co-produced with older prisoners and ex-prisoners could explore, adapt, and modify this framework, including reflection on the application of existing frameworks, so as to design and pilot a tool specifically for assessing the age-friendliness of cities and communities for prisoners, ex-prisoners and, indeed, for older people serving community penalties such as probation. This holistic, co-produced approach could gather data about older peoples’ lived experiences during and after release from custody, and also prioritise the aspects of community re-entry and integration which are most important to older people, rather than focusing solely on the demands of offender management. This approach needs to adopt a realist approach, recognising that older people do not all reflect the characterisation of the positive, community-engaged older person as portrayed in the research literature, and recognising the nuances and dynamics of seeking age-friendliness. For example, whilst it is in many ways desirable to create opportunities for inter- and multi-generational contact, as exemplified in projects which build care homes and nurseries on the same site, safeguarding controls would mean that an exclusionary approach would have to be adopted for some older people if they have offended against children. A key question then is of how to create and enable beneficial inter- and multi-generational contacts within a framework of safeguarding. Conversely, some young people may themselves pose a risk to vulnerable older people, and thus again questions of how to manage inclusion come to the fore.

Buckner et al. [62] postulate that “it is the role of cities with an age-friendly agenda to create environments where higher-level influences interact with local-level policies and action in such a way as to foster active ageing and living as well as possible in older age”. Some of the research literature refers to inclusion and marginalisation of some groups, including disabled older people, and a key challenge to the notion of an age-friendly city is the question of how the city includes people whom others may not want to be included, or who are socially, economically and politically marginalised. These issues go beyond questions of urban planning to become broader questions of social justice. For a city to be genuinely age-friendly it needs to be age-friendly for all older people, including those who experience imprisonment.
